# Methodology for LoRa Gateway Placement Based on Bio-Inspired Algorithmsfor a Smart Campus in Wooded Area

**DOI:** 10.3390/s22176492

**Published:** 2022-08-29

**Authors:** Hugo A. O. Cruz, Sidnir C. B. Ferreira, Jasmine P. L. Araújo, Fabrício J. B. Barros, Fabrício S. Farias, Miércio C. A. Neto, Maria E. L. Tostes, Andréia A. Nascimento, Gervásio P. S. Cavalcante

**Affiliations:** 1 Electrical Engineering Pós-Graduate Department, Federal University of Pará, Belém 66075-110, Brazil; 2 Information Systems Department, Federal University of Pará, Cametá 68400-000, Brazil; 3 Research and Development Program, Norte Energia S.A, Brasília 70714-900, Brazil

**Keywords:** EPSO, IoT, LoRa, LPWANs, smart campus, gateway placement, propagation model

## Abstract

The Internet of Things (IoT) device scenario has several emerging technologies. Among them, Low-Power Wide-Area Networks (LPWANs) have proven to be efficient connections for smart devices. These devices communicate through gateways that exchange points with the central server. This study proposes an empirical and statistical methodology based on measurements carried out in a typical scenario of Amazonian cities composed of forests and buildings on the Campus of the Federal University of Pará (UFPA) to apply an adjustment to the coefficients in the UFPA propagation model. Furthermore, an Evolutionary Particle Swarm Optimization (EPSO) metaheuristic with multi-objective optimization was applied to maximize the coverage area and minimize the number of gateways to assist in the planning of a LoRa network. The results of simulations using the Monte Carlo method show that the EPSO-based gateway placement optimization methodology can be used to plan future LPWAN networks. As reception sensitivity is a decisive factor in the coverage area, with −108 dBm, the optimal solution determined the use of three gateways to cover the smart campus area.

## 1. Introduction

The fourth industrial revolution, combined with the rapid development of information and communication technologies, established a new scenario in which automation is no longer static and relies on equipment and systems connected to the internet. This brings efficiency to various processes, enabling the introduction of new technologies and strengthening the paradigm known as the Internet of Things (IoT). Some new scenarios are presented through emerging concepts, such as smart cities and smart campuses, with the IoT being strongly present in both [1]. Practically, the IoT enables the deployment of intelligent sensor networks that allow the automation of mobility systems [2,3], better energy efficiency [4], rapid monitoring of health data [5], intelligent management of solid waste collection [6], etc., which are directly linked to the specificities and daily life of the scenarios in question.

Currently, several wireless communication technologies can be applied to IoT systems and are grouped into short, medium, and high distances. The first depends on the data rate and coverage and can be based on Bluetooth, Zigbee, and Wireless Fidelity (Wi-Fi) technologies, with the ability to serve users arranged in local short-distance networks [7]. The second wireless alternative is based on cellular networks, aimed at devices that require high data rates at a medium distance [8]. In this case, Heterogeneous Networks (HetNets) are composed of macro, micro, pico, and femtocells to provide indoor and outdoor coverage for users served by the [9] service. Finally, there is a category of wireless technologies for Long-Range (LoRa) networks and Low-Power Wide-Area Networks (LPWAN), which offers communication over long distances and low power consumption at the expense of low data rate usage [10].

LPWANs can use the LoRa technology for communication. LoRa is a promising air interface alternative that operates at an unlicensed frequency range below 1 GHz. In addition, LoRa offers a long-range communication link with low power consumption and high reliability, and communication is established through the communication protocol LoRa Wide-Area Network (LoRaWAN), standardized by the LoRa Alliance [11]. Its topology is organized as a star and allows the retransmission of messages between the end devices and a central network through gateways that aggregate the data transmitted by the network [12].

**Figure 1:**
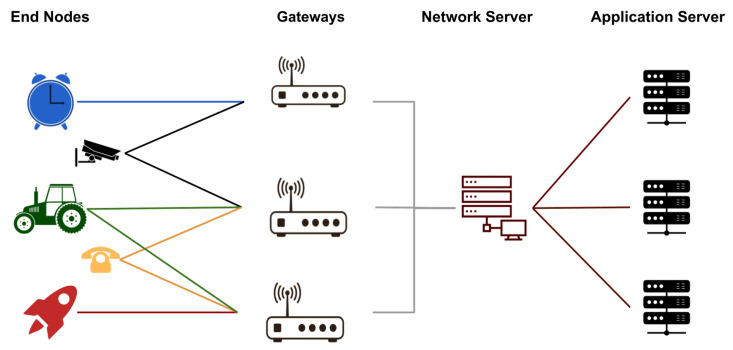
Topology of a LoRa network [12].

Gateways are the elements responsible for providing communication between the end devices (end nodes) and the network server, which communicates with the application server, as shown in Figure 1. For such communication to occur, the gateways need to be strategically located in the scenario and in sufficient quantity to offer optimal coverage so that all sensors can be connected and transmit information in real time [12].

Several authors have investigated the technology from different points of view, including the communication LoRa [11], communication protocol [13], coverage radius [14,15], intensity level [16], mobility [17,18], channel modeling [19,20], and gateway placement [21,22].

In [11], the coverage of LoRa communication at the State University of Campinas (UNICAMP) was analyzed to assess the feasibility and reliability of real-time control and monitoring systems. The authors performed several tests by considering different distances, modulation settings, and packet sizes. However, no planning was carried out to determine the best location for installing the gateway, which is a necessary step to avoid errors, reduce costs, and optimize network deployment. In addition, only one gateway was used in the tests, which may not guarantee optimal coverage at specific locations.

The study in [13] analyzed the LoRaWAN protocol based on architecture, battery life, network capacity, device classes, and security. The results demonstrated that LoRaWAN can provide coverage of 2–5 km in urban areas and up to 45 km in rural areas, reaching data rates of 290 bps and 50 kbps, respectively. However, these data are only theoretical and do not consider the morphology of the environment or the possible impacts that may occur in the coverage area as a result of obstacles in the communication channel. According to [23], these factors are crucial for obtaining a realistic analysis.

In [14], the coverage of a LoRa network was studied through measurements carried out in the city of Oulu, Finland, for a frequency band of 868 MHz and transmission power of 14 dBm. In this investigation, the maximum propagation factor was found with a transmission range of more than 15 km to the receiver on the ground (connected to the roof of a car) and 30 km in the water (connected to the mast of a boat).

In [15], the propagation of LoRa signals in forest, urban, and suburban vehicular environments was discussed. In addition to environments with variable propagation conditions, scenarios with node mobility were evaluated. To characterize the signal propagation, the Received Signal Strength Indicator (RSSI), Signal-to-Noise Ratio (SNR), and Packet Delivery Ratio (PDR) were used. The results showed that the link reached up to 250 m in the forest scenario. In scenarios with high-density buildings and human activity, the maximum reach of the link was 200 m in the urban scenario.

The results of [14,15] show the impact of the environment on the coverage area, which must be considered in the planning stage to provide maximum coverage.

The authors in [16] evaluated the level of signal strength received by a LoRaWAN network deployed in Skellefteå, Sweden. The obtained data were compared with simulations of a radiofrequency planning tool using Irregular Terrain Propagation (ITM), Irregular Terrain With Obstruction (ITWOM), and Okumura–Hata propagation models. The results of the ITWOM model were closer to the collected data. Furthermore, it was found that the choice of the propagation model must be careful and must always meet the environmental conditions. This restriction must be considered when defining the objective function proposed in this study.

Another aspect that should be considered in the study of LoRa technology is mobility, which was analyzed in [17], and the performance of a LoRaWAN network (915 MHz) in indoor and outdoor environments, taking into account different degrees of mobility, with end-to-end delay and Packet Loss Rate (PLR) as the main parameters.

In [18], a comparison was made between the real and simulated scenarios (NS-3), referring to a LoRaWAN network (915 MHz) for vehicular communication (mobility), aiming to analyze and compare the performance in both cases by considering three measures, namely, PDR, Packet Intercept (PIR), and RSSI. The experiments were conducted at the Federal University of Rio de Janeiro (UFRJ) and consisted of traveling a route of 1 km at speeds of 30 km/h and 60 km/h with SF values of 7, 9, and 12. The results in [17,18] indicate that the mobility significantly increased the delay, as well as the speed and distance traveled.

To establish a greater accuracy of the propagation models, adjustments are made to adapt the average behavior of the propagated signal to the measured data in specific environments. Some works use bio-inspired optimization techniques. In [19], the Genetic Algorithm (GA), Flower Pollination Algorithms (FPA), and Bat Algorithm (BAT) are used to adjust for LTE frequency range in the Amazon region which is densely wooded. In [20], Particle Swarm Optimization (PSO) is used to optimize the fitting curve parameters to the models generated from mathematical regression techniques for an alfalfa plantation environment applied to ZigBee technology for smart agriculture.

Other works were developed to optimize the gateway placement for the LoRa network, as in [21,22], which uses network simulation software (NS-3 simulator) to evaluate performance based on Quality of Service (QoS) metrics. Both works seek to optimize gateways allocation in area X and can post them anywhere within that area by applying classical propagation models.

On the other hand, the proposed methodology optimizes the gateways placement points from pre-established locations by a network planning specialist, imposing a restriction to make the process less computationally expensive, preventing the algorithm from choosing an inappropriate place physically, environmentally, and financially, keeping in mind that we are dealing with a wooded region, which is difficult to access.

In addition to performing an adjustment in a regional propagation model through a GA based on measurements performed in the place where the implementation of the IoT network is desired, we also intend to improve the accuracy in the positioning of the gateways to serve the entire coverage area of the smart campus, ensuring a quality signal using SF7 and establishing a conservative communication threshold, that is, ensuring uninterrupted communication with all devices within the smart campus.

Thus, this study aims to estimate the signal level of a LoRa network using data measured at the Belém Campus, Federal University of Pará (UFPA), a typical highly wooded Amazonian environment, applying an empirical model called UFPA for prediction of the RSSI adjusted through a Genetic Algorithm (GA). To determine the signal coverage radius, define the best positioning of the gateways, maximize the coverage area, and minimize the amount of equipment needed to implement a LoRa network, was used the Evolutionary Particle Swarm Optimization (EPSO) multi-objective optimization technique.

Section 2 describes the signal strength measurement campaign conducted on the campus. The methodology used is described in Section 3. In Section 4, the results and a discussion are presented. Finally, Section 5 presents the final considerations of the study and discusses future work.

## 2. Measurement Campaign

The measurements aimed to obtain data describing the propagated signal’s average behavior in a specific environment. Data regarding RSSI values and geolocation were collected through devices with LoRaWAN technology, which were used to readjust an empirical model of propagation to minimize the RSSI prediction error and consequently define the positioning of the gateways with greater precision. This section describes measurements, the scenario, equipment, and measurement methodology.

### 2.1. Scenario

**Figure 2:**
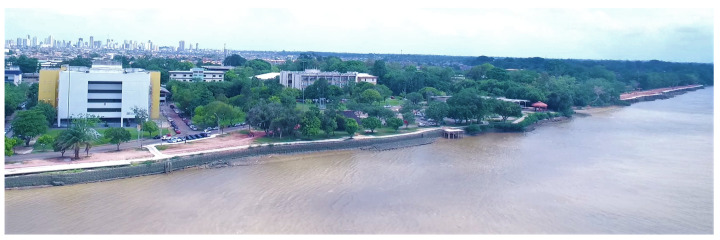
UFPA Campus.

The measurements were performed at the Federal University of Pará (UFPA) campus in Belém, Pará, Brazil. UFPA has an environment composed of buildings and trees and is located on the banks of the Guamá River, a typical Amazonian scenario, as shown in Figure 2, which illustrates the characteristics of the environment where the measurement campaign (in this case, wooded) was carried out to develop the work.

### 2.2. Used Equipment

**Figure 3:**
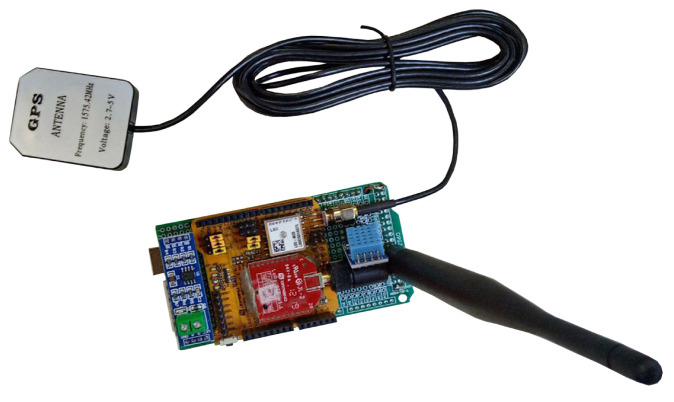
SX1276 module coupled to an Arduino UNO.

For this experiment, two Dragino model SX1276 radio modules were used, equipped with the L80-m39 GPS module, each connected to an Arduino UNO and equipped with omnidirectional antennas with a gain of 1 dBi, as illustrated in Figure 3. It is noteworthy that each pair of devices was connected to a laptop, with one serving as a transmitter, and the other as a receiver.

During the experiments, the devices were configured with respect to their operating frequency, effective radiated power, SF, bandwidth, and coding rate, and the values are presented in Table 1.

### 2.3. Measurements

The measurement methodology was based on the ability of the device under investigation to receive information from various devices spread across the campus.

**Figure 4:**
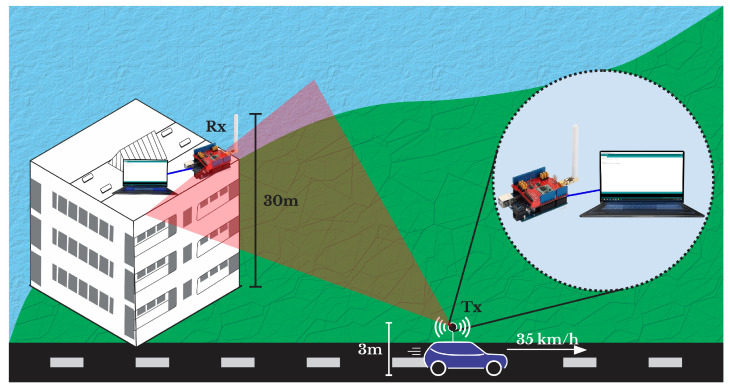
Measurement scheme.

Two sets of equipment were used to collect the measured data, consisting of an SX1276 module coupled to an Arduino fixed at a height of 3 m and a laptop as a transmitter (Tx), which moved along the established route with an average speed of 35 km/h. The other set of equipment was configured as a receiver (RX), positioned on top of the “Mirante do Rio” building, which is installed 30 m above sea level, as shown in Figure 4, to capture the signal with the message containing the latitude, longitude and RSSI values, accounting for a total of 128 kbit. The measurements were performed by considering the uplink of the signal (car to building), and this situation represents the worst channel condition to the detriment of the downlink (building to car).

The data collected were composed of the RSSI value and its geographic coordinates, considering the randomness of the signal in the scenario with mobility, and were measured five consecutive times on the same route. An average of 246 samples per measurement were obtained, totaling 1230 measurements, with messages received in short intervals of 4.5 s or medium distances of up to 43.74 m (for a car traveling at 35 km/h).

## 3. Methodology

**Figure 5:**
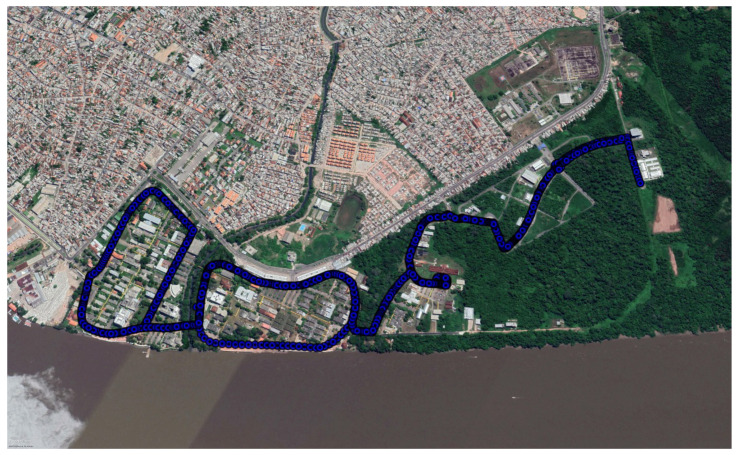
The route to be covered by the LoRaWAN network is represented in blue.

This section will approach the methodology adopted in the study to define the best locations for gateway installations to obtain maximum coverage with the least number of gateways. To monitor the electric bus and its parameters (i.e., location, level, and temperature of the vehicle’s batteries), a survey of the geographic data of latitude and longitude of the route, 2.5 km along the campus, was conducted, as illustrated in Figure 5.

For this purpose, measurements were carried out with GPS in which 957 geolocations were verified, which stipulate the route to be served by the LoRa network that will be implemented.

**Figure 6:**
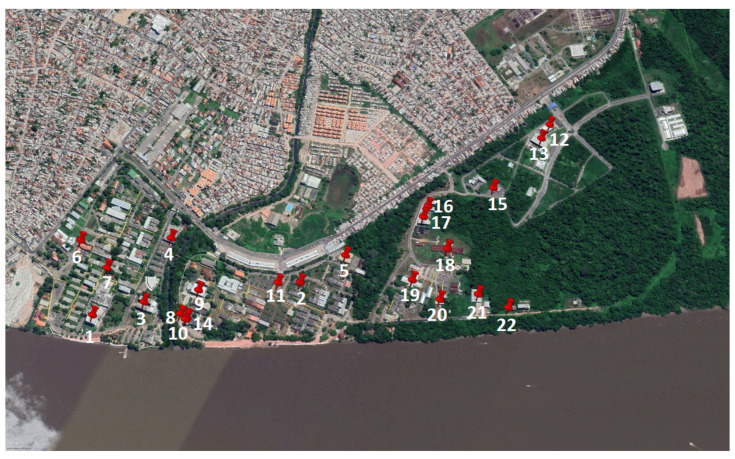
Candidate points for installing the gateways are represented in red.

Therefore, a technical feasibility analysis was carried out to install gateways at strategic points on the campus, considering the infrastructure to meet the needs of electrical installation and connection to the database server via the Internet. Thus, the positions of the 22 candidate points were defined for the possible installation of the gateways, as shown in Figure 6.

After defining the candidate points, the following data pre-processing steps were applied. The propagation losses and distance between the transmitter and receiver were calculated. Next, each step of the methodology was described, along with the adjustment and application of the UFPA propagation model to approximate the measured and predicted data. Then, a statistical analysis of the coverage threshold was conducted, and the EPSO technique was used to define the best locations for the gateways. Finally, Monte Carlo simulations were performed.

### 3.1. Data Pre-Processing

After obtaining the data in the measurement stage, it was necessary to process them to obtain two critical metrics for the development of the work: received power and distance between the transmitter and receiver.

#### 3.1.1. Calculation Distance between Transmitter and Receiver

To calculate the distance between the transmitter and the receiver, the values of latitude and longitude captured were inserted into the Haversine equation (Equation(1)) as an input parameter for the loss model of propagation [24].

(1) $$\begin{array}{c} d=2r\arcsin\left(\sqrt{\sin^{2}\left(\frac{\varphi_{2}-\varphi_{1}}{2}\right)+\cos\left(\varphi_{1}\right)\cos\left(\varphi_{2}\right)\sin^{2}\left(\frac{\lambda_{2}-\lambda_{1}}{2}\right)}\right), \end{array}$$

where *d* is the distance between two points in meters; *r* is the radius of the earth in meters (6,371,000 m); $\varphi_{1}$ and $\varphi_{2}$ represent the latitudes of point 1 and point 2, respectively; $\lambda_{1}$ and $\lambda_{2}$ represent the longitudes of point 1 and point 2, respectively.

#### 3.1.2. Calculation of Received Power

The received power level was calculated for the entire route and analyzed for each candidate point for installation (Figure 6). To obtain the power level, propagation models can be applied to predict the signal Path Loss (PL), which, when subtracted from the transmitted power, has the received power of the signal at a given point, according to (2):

(2) Pr=Pt+G−PL

where Pr is the power received at a given point in dBm; Pt is the power transmitted by the gateway in dBm; *G* is the sum of transmission and reception gains, usually in dBi; PL represents the signal propagation loss in dB, predicted by a given propagation model.

### 3.2. UFPA Propagation Model

For the development of the research, the empirical propagation model called UFPA [25] was selected, which consists of several measurements carried out in the Amazonian region and is represented by (3).

(3) $$PL=K_{1}\log_{10}(d)+K_{2}\log_{10}\left(f\right)+K_{0},$$

where *d* is the distance between the transmitter and receiver in meters, *f* is the frequency in MHz, $K_{1}$ and $K_{2}$ are adjustable parameters according to the environment, and $K_{0}$ is a correction factor expressed by (4):

(4) $$K_{0}=a-bX.$$

Parameters *a* and *b* expressed in (4) are also obtained using the linear least squares technique, and factor *X* is calculated from (5).

(5) $$X\;=\;\frac{\left(Ht+\;Hr\right)\lambda \;}{\left(0.1\;*\;H_{OB}\right)}$$

where Ht and Hr are the heights of the transmit and receive antennas, respectively; λ is the wavelength; $H_{OB}$ is the average of the ambient obstructions.

The model is used to estimate the signal coverage radius with the aid of (2) to establish reliable communication, thus replacing the received power with the sensitivity threshold in dBm. Therefore, it is necessary to adjust the parameters of the propagation model and define the threshold through a statistical analysis based on the data obtained during the measurement campaign.

### 3.3. Adjustment of the UFPA Model through Genetic Algorithm (GA)

Empirical propagation models are based on extensive measurement campaigns to establish the average behavior of the propagated signal in a given environment, considering parameters that influence signal propagation such as distance and height between transmitters and receivers, frequency of operation, etc.

Each model has an operating range determined for each parameter based on the measurements carried out in its design. This way, a model operates with a minimum and maximum distance and height and in a specific frequency range. Another aspect that should be highlighted is the path loss exponent, which indicates that the signal decay factor in certain types of environments (urban, suburban, rural) varies according to the morphological characteristics of the studied environment.

Therefore, adjustments may be needed to use a specific model because it is outside a particular parameter established in its design.

The chosen path loss model operates at a frequency of 5.8 GHz. To achieve this, adjustments are necessary in the frequency range of 5.8 Ghz to 915 MHz. Using the data obtained in the measurements, distance values, and RSSI, it was possible to optimize parameters “k1’’, “k2’’, “a’’, and “b’’ in Equation (3). The bio-inspired GA technique was implemented to minimize the error between the UFPA propagation model and the measured data. That way, it was adjusted to follow the trend of the measured data, thus making the positioning of the gateways more accurate [19].

#### 3.3.1. UFPA Model Adjustment Objective Function

To adjust the model, the Root Mean Squared Error (RMSE) was defined as the objective function, which calculates the difference between the predicted and measured values using (6), where *n* is the number of samples, Mv is the measured value, and Pv is the predicted value for the *i*-th sample.

(6) $$RMSE=\sqrt{\frac{\sum_{i=1}^{n}{\left(M_{vi}-P_{vi}\right)}^{2}}{n}}$$

#### 3.3.2. Description of the Implemented Genetic Algorithm

The adjustment was performed using the GA, which is an optimization technique that employs iterative algorithms that simulate the evolution process of a given population, with each individual in this population representing a possible solution to the problem.

However, the evolution process is random, guided by a selection mechanism based on the adaptation of individual structures. With each generation of individuals, a new set of structures is developed by exchanging information between the best-adapted individuals of the previous generation.

As a result of this process, it is noted that the fittest individuals continue to share their structures, which provides an increase in fitness with each generation. Thus, there is an approximation of an optimal solution.

Table 2 explains the parameters used in the implemented GA:

### 3.4. Coverage Threshold Analysis

**Figure 7:**
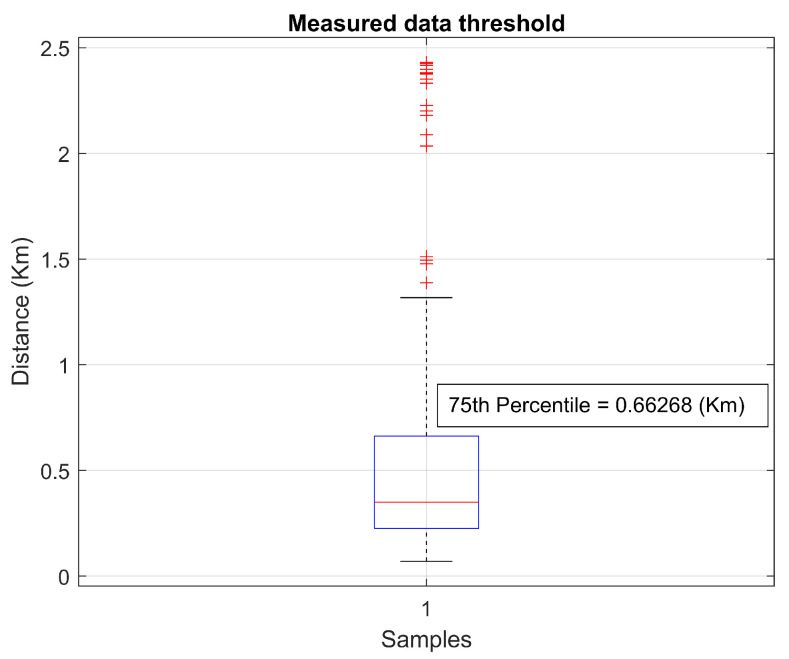
Statistical analysis of coverage radius.

The signal coverage radius is estimated from a statistical analysis of the data collected in the measurements referring to distances, as illustrated in Figure 7.

Therefore, quartiles are values extracted from a set of data observed in ascending order and divide the distribution into four equal parts. The first quartile (Q1) corresponds to the first 25% of the data; the second quartile (Q2) corresponds to the range between 25 and 50% (median); the third quartile (Q3) corresponds to the range between 50 and 75%; the fourth quartile (Q4) corresponds to the ranges between 75 and 100%.

In this context, the 75th percentile value of 0.66268 km or 662.68 m was defined to determine the coverage radius threshold for a mobile object uplinking data between end nodes and the gateway to ensure reliable communication to the proposed link. For values greater than the defined, the signal becomes non-continuous, for smaller values, it would be a very conservative coverage radius.

### 3.5. Gateway Optimization

#### 3.5.1. Gateway Optimization Purpose Function

The objective function defined in this study for EPSO is multi-objective, as it searches to minimize the uncovered area (or maximize the covered area) and the number of gateways to reduce implementation costs. The minimization of the uncovered area is given by Obj1, as follows:

(7) $$Obj1=\left(At-Pc\right)$$

In (7), At is the total area to be covered (all 957 points that make up the route to be served) in percentage (100%) and Pc is the coverage index in percentage (the number of points served, that is, with the RSSI value greater than or equal to the established threshold of −108 dBm).

ObJ2 minimizes the number of gateways required, as follows:

(8) $$Obj2=\;\frac{N_{g}}{N_{gt}}$$

where $N_{g}$ represents the number of gateways defined as optimal by EPSO and $N_{gt}$ is the total number of candidate gateways.

The development of the multi-objective function was based on the concept known as Pareto optimal front [26]. This concept seeks a balance between two or more goals.

Equations (9) and (10) represent the calculations for defining the optimal Pareto fronts, where *W* is the average value of the *N* Pareto solution for *i* iterations of the algorithm and *Z* is the final equation of the multi-objective function.

(9) $$W=\frac{i}{N}$$

(10) Z=Obj1∗W+(1−W)∗Obj2

It is worth mentioning that the objective function seeks to minimize the number of gateways and the area not covered, with a Pareto front ranging from 1 to 10. As the Pareto front increases, coverage is prioritized over minimizing the number of gateways. The higher the *W* value, the more gateways will be used to reduce the area not covered. Conversely, smaller values of *W* result in fewer gateways, reducing deployment costs without guaranteeing the maximum possible coverage.

#### 3.5.2. Description of EPSO Implemented

The optimization model implemented in the simulations is the Evolutionary Particle Swarm Optimization (EPSO), which is based on evolutionary simulations [27] and Particle Swarm Optimization algorithms [28], thus resulting in a self-adaptive evolutionary algorithm, in which a particle motion operator replaces the recombination. The new operator proves to be more effective than the recombination in generating solutions that approach the optimal value. Furthermore, its self-adapting feature makes EPSO independent of the external definition weights and parameters in [29].

EPSO’s operating scheme follows five steps for a given iteration: replication, mutation, reproduction, evaluation, and selection. EPSO’s motion rule is for a particle $X_{i}$, a new particle $X_{i}^{new}$ is given by [30]:

(11) $$X_{i}^{new}=X_{i}+V_{i}^{new},$$

and their respective speeds are

(12) $$V_{i}^{new}=w_{i0}^{*}V_{i}+w_{i1}^{*}\left(b_{i}-X_{i}\right)+w_{i2}^{*}\left(b_{g}^{*}-X_{i}\right).$$

The motion rule retains its terms of inertia, memory, and cooperation, as in the standard PSO algorithm. However, in EPSO, the weights change as (13):

(13) $$w_{ik}^{*}=w_{ik}+\tau N\left(0,1\right),$$

where N(0,1) represents a random variable with a Gaussian distribution, mean 0, and variance 1. The global best $b_{g}$ is randomly perturbed, as follows:

(14) $$b_{g}^{*}=b_{g}+\tau^{\prime }N\left(0,1\right).$$

τ and $\tau^{\prime }$ are fixed learning parameters or are treated as strategic parameters and, therefore, subject to mutation.

Table 3 consolidates the strategic parameters defined for EPSO, which are added in (13) and (14).

### 3.6. Monte Carlo Simulation

Monte Carlo simulations are based on probabilistic and stationary approaches [31]. This method can be used together with other methods to solve probabilistic problems, such as server positioning in [32] and network topology projects and reliability testing in [33].

This study analyzes the stability and reliability of EPSO’s applied technique in searching for results. The most frequent set of solutions was chosen through 300 Monte Carlo simulations executed for each Pareto front. The next section presents and discusses the results.

## 4. Results

In this section, the results obtained during the study are presented, including the data collected during the measurement campaign, adjustment of the UFPA propagation model using the genetic algorithm technique, and evaluation of the model’s predictive capacity before and after the adjustment. In addition, the need for accuracy and precision of the model for the implementation of the IoT network, as well as the issue of multi-objective optimization through EPSO to determine the optimal points for gateway installations through Monte Carlo simulations, are discussed.

### 4.1. Analysis of Measured Data

**Figure 8:**
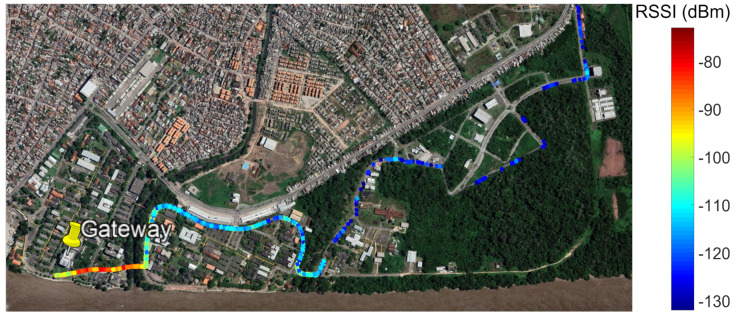
Route with measured data with location and RSSI value.

Figure 8 shows the data collected during the measurement campaign with RSSI values ranging from −80 to −130 dBm with their respective geographic location on the map. Thus, to reduce the signal attenuation effects caused by the wooded environment under analysis, the Pareto 8 front was selected as the most suitable for the scenario, prioritizing the maximization of the coverage area and minimizing the shading effects caused by forests with fewer gateways.

### 4.2. Adjusted Model

Therefore, applying the standard parameters of the UFPA model k1 = 16.5, k2 = 14.2, a = 42.5, and b = 7.6, the RMSE value between the measured and predicted data of 26.68 dBm was obtained. The parameters after optimization were k1 = 28.39, k2 = 29.13, a = 9.86, and b = 12.14, generating an RMSE between the measured and predicted data with the UFPA model adjusted to 9.00 dBm.

**Figure 9:**
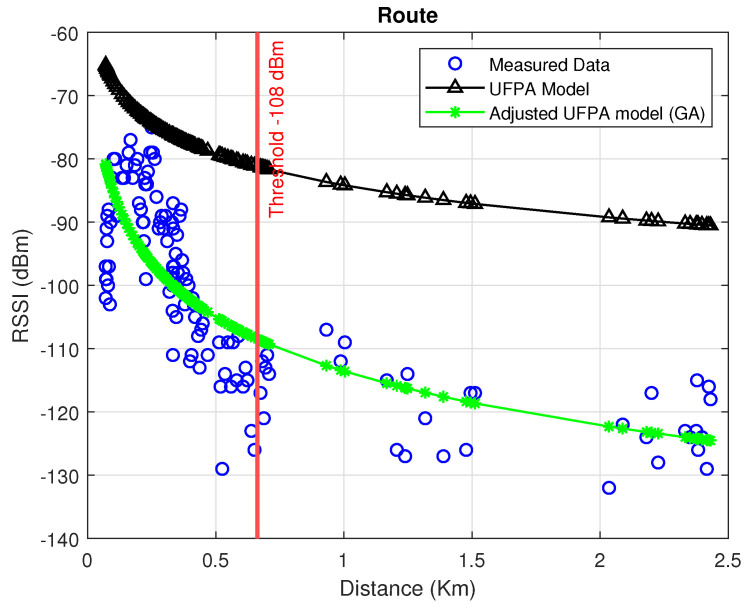
Comparison between measured data and models.

Figure 9 displays the data collected in the measurement campaign, those predicted by the UFPA model, and those predicted by the adjusted UFPA model, in addition to displaying the value of the power threshold of −108 dBm, which is associated with the distance threshold of 0.66862 km for the case studied.

### 4.3. Gateway Optimization

The simulations showed that the propagation model and equipment sensitivity significantly affected the number of gateways needed and their positions. Therefore, it is necessary to use a specific propagation model for the deployment scenarios. Furthermore, the sensitivity must be configured according to the project needs such that the planning simulations have better accuracy.

**Figure 10:**
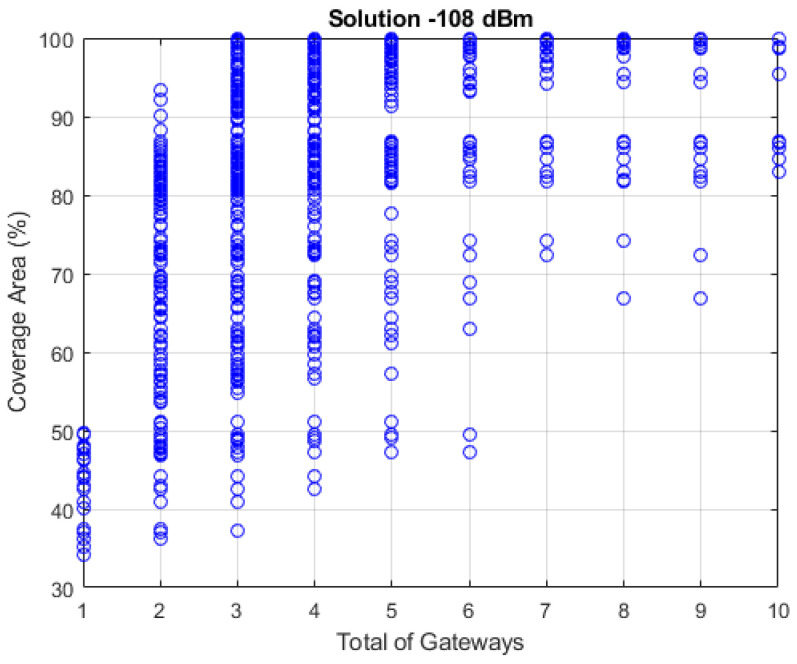
Solutions for −108 dBm sensitivity.

In Figure 10, all the optimal points are presented at each generation for the threshold of −108 dBm, taking into account the 300 simulations at 10 Pareto fronts and 50 generations, totaling 150,000 solutions; the y-axis represents the covered area values in relation to the number of gateways ranging from 1 to 10 on the x-axis.

Note that the covered area for solutions with one gateway is approximately 50% in the best cases. For the two gateways, it is approximately 95%, while for the three gateways, the covered area is fully maximized, with EPSO achieving a solution capable of serving 100% of the established route. Thus, the most suitable solution for implementing the LoRa network is to use three gateways because it can cover 100% of the route with the smallest number of gateways.

**Figure 11:**
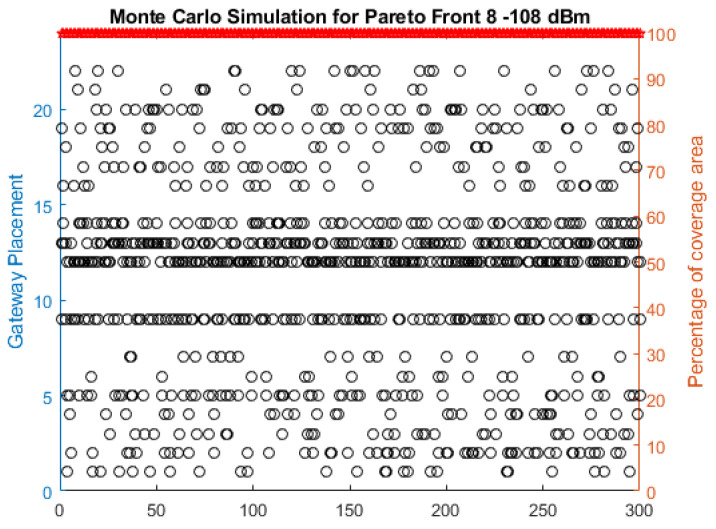
Monte Carlo simulation result for Pareto front and −108 dBm.

In Figure 11, the results of the simulations obtained through the EPSO technique are presented, applying the Monte Carlo method for the Pareto front 8 and a threshold of −108 dBm, in which the optimal solutions obtained for each of the 300 simulations. The x-axis represents the number of simulations performed, the left y-axis represents the gateway position, and the right axis represents the percentage of the covered area. The black circles indicate the solutions found by EPSO, and the red stars indicate the percentage of the coverage area resulting from these solutions.

**Figure 12:**
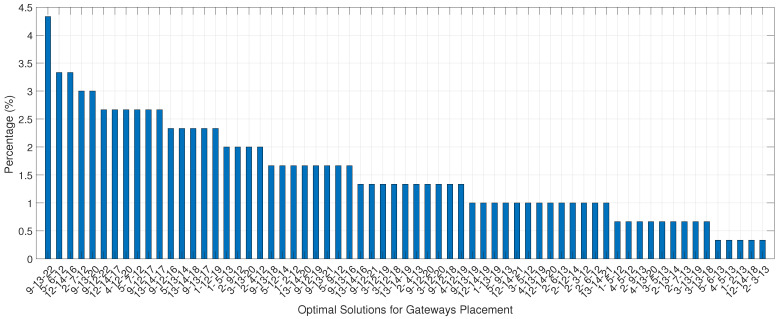
Frequency of Pareto 8 solutions with −108 dBm sensitivity.

Figure 12 illustrates the frequency of occurrence of optimal solutions for the threshold of −108 dBm for the Pareto 8 front as a function of the number of simulations. The most frequent solution is composed of three gateways, 9- 13-22, and it occurred in 4.33% of the 300 simulations, from which 65 different optimal solutions were obtained. Each value of the most frequent solution is represented by its respective location using a pair of latitude and longitude coordinates, as illustrated in Figure 6.

The other 64 solutions presented for locating gateways can also serve 100% of the area to be covered with only three gateways. However, they were not selected because they were less frequent. Nevertheless, depending on the implementation cost, they can also be considered.

**Figure 13:**
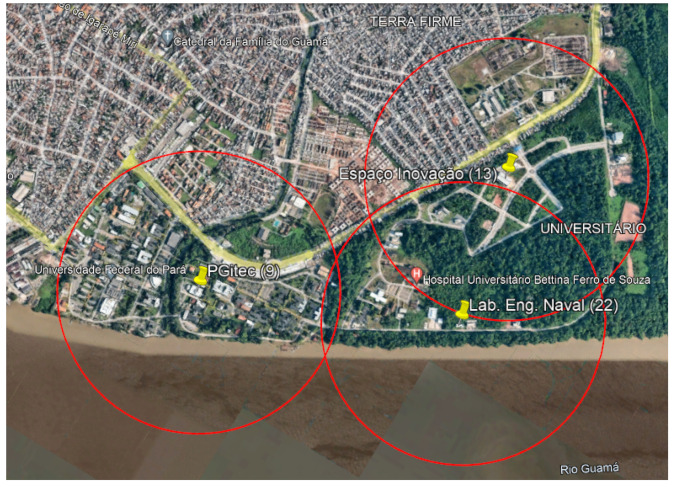
Optimal gatewayplacement for sensitivity of −108 dBm.

The positioning of the gateways for the optimal solution with a sensitivity of −108 dBm using the Pareto 8 front is illustrated in Figure 13. The yellow dots are the optimal locations of the gateways in positions 9-13-22, and the red circles represent the coverage radius of each gateway estimated by the UFPA model, adjusted to 662.68 m, which provides 100% coverage.

When developing an IoT network for scenarios where communication must be reliable and have low latency, the received signal power must be prioritized. Therefore, it is crucial to define a higher reception sensitivity that reduces the coverage area. In this case, the number of gateways must be increased until the required total coverage area is achieved. However, for cases in which coverage must be prioritized or implementation costs must be reduced to the detriment of signal quality, it is possible to work with lower sensitivity, thus attaining greater coverage distances by equipment.

## 5. Conclusions

The objective of this study was to develop a multi-disciplinary methodology capable of maximizing the coverage area and minimizing the number of gateways needed to implement a LoRa network at the Belém Campus, at the Federal University of Pará, in a typical highly wooded Amazonian environment, considering the particularities of the scenario studied, such as the fact that it is separated by forests and streams, which causes the signal to suffer strong attenuation.

Therefore, it is necessary to associate several areas of knowledge as instrumentation for measurements of electromagnetic wave signals to obtain RSSI values in the studied environment. Propagation was essential for developing the fit of the UFPA propagation loss model, performed through GA and multi-objective bio-inspired optimization to establish the maximum coverage with the least number of gateways. Additionally, the simulations were validated through statistical means using the Monte Carlo method.

Statistical analysis determined that a sensitivity threshold of −108 dBm was adequate to cover the proposed environment, obtaining an estimated radius with the adjusted UFPA model of 668.62 m. The optimal solution found using the EPSO technique for positioning LoRa gateways was to establish three gateways positioned at candidate points 9-13-22, providing 100% coverage for the studied environment. This demonstrates that the proposed methodology can assist in the future planning of IoT networks by reducing costs and possible deployment errors using a suitable propagation loss model for each region to be implemented.

Finally, in future work, the application will be expanded to analyze the positioning of gateways for the city of Belém and the implementation of other metaheuristic techniques to statistically analyze the best approach for the optimal positioning of gateways for IoT LoRaWAN network planning.

## Figures and Tables

**Table 1:** Device configuration parameters used.

Parameter	Value
Operating frequency:	915 MHz
Effective radiated power:	20 dBm
Spreading factor:	7
Bandwidth:	125 KHz
CodingRate:	5(4/5)

**Table 2:** Genetic Algorithm Parameters.

Parameter	Value
Initial Population:	50
Population Size:	50
Elite Count:	0.05
Crossover Fraction:	0.8
Stopping Criteria: Generations/Stall generations:	500/50
Intermediate Crossover Function	
Gaussian Mutation function	
Rank Scale Function	

**Table 3:** Strategic parameters of EPSO.

Parameter	Value
Population size	100
Mutation rate	0.4
Communication probability	0.7
Max. number of fitness evaluations	10,000
Max. number of generations	100,000
Max. number of generations with the same global best	100

## Data Availability

The authors reserve the right to not disclose the private data set used in this study.
